# Bisdemethoxycurcumin Induces Cell Apoptosis and Inhibits Human Brain Glioblastoma GBM 8401/*Luc2* Cell Xenograft Tumor in Subcutaneous Nude Mice In Vivo

**DOI:** 10.3390/ijms23010538

**Published:** 2022-01-04

**Authors:** Te-Chun Hsia, Shu-Fen Peng, Fu-Shin Chueh, Kung-Wen Lu, Jiun-Long Yang, An-Cheng Huang, Fei-Ting Hsu, Rick Sai-Chuen Wu

**Affiliations:** 1 Department of Respiratory Therapy, China Medical University, Taichung 406, Taiwan; D1914@mail.cmuh.org.tw; 2 Department of Internal Medicine, China Medical University Hospital, Taichung 404, Taiwan; 3 Department of Medical Research, China Medical University Hospital, Taichung 404, Taiwan; t20811@mail.cmuh.org.tw; 4 Department of Biological Science and Technology, China Medical University, Taichung 406, Taiwan; 5 Department of Food Nutrition and Health Biotechnology, Asia University, Taichung 413, Taiwan; fushin@asia.edu.tw; 6 School of Post-Baccalaureate Chinese Medicine, College of Chinese Medicine, China Medical University, Taichung 406, Taiwan; prorna@mail.cmu.edu.tw; 7 Department of Nursing, St. Mary’s Junior College of Medicine, Nursing and Management, Yilan 266, Taiwan; yangjiunlong@gmail.com (J.-L.Y.); haj@smc.edu.tw (A.-C.H.); 8 Department of Anesthesiology, China Medical University Hospital, Taichung 404, Taiwan; 9 Department of Anesthesiology, China Medical University, Taichung 404, Taiwan

**Keywords:** BDMC, glioblastoma (GBM) 8401/*luc2* cells, apoptosis, xenograft, BAX, Bcl-2

## Abstract

Bisdemethoxycurcumin (BDMC) has biological activities, including anticancer effects in vitro; however, its anticancer effects in human glioblastoma (GBM) cells have not been examined yet. This study aimed to evaluate the tumor inhibitory effect and molecular mechanism of BDMC on human GBM 8401/*luc2* cells in vitro and in vivo. In vitro studies have shown that BDMC significantly reduced cell viability and induced cell apoptosis in GBM 8401/*luc2* cells. Furthermore, BDMC induced apoptosis via inhibited Bcl-2 (anti-apoptotic protein) and increased Bax (pro-apoptotic proteins) and cytochrome c release in GBM 8401/*luc2* cells in vitro. Then, twelve BALB/c-nude mice were xenografted with human glioblastoma GBM 8401/*luc2* cancer cells subcutaneously, and the xenograft nude mice were treated without and with BDMC (30 and 60 mg/kg of BDMC treatment) every 3 days. GBM 8401/*luc2* cell xenografts experiment showed that the growth of the tumors was significantly suppressed by BDMC administration at both doses based on the reduction of tumor size and weights. BDMC did not change the body weight and the H&E histopathology analysis of liver samples, indicating that BDMC did not induce systemic toxicity. Meanwhile, treatment with BDMC up-regulated the expressions of BAX and cleaved caspase-3, while it down-regulated the protein expressions of Bcl-2 and XIAP in the tumor tissues compared with the control group. This study has demonstrated that BDMC presents potent anticancer activity on the human glioblastoma GBM 8401/*luc2* cell xenograft model by inducing apoptosis and inhibiting tumor cell proliferation and shows the potential for further development to the anti-GBM cancer drug.

## 1. Introduction

Glioblastoma (GBM) in adults and medulloblastoma and pineoblastoma in children are aggressive brain tumors. GBM is the fatal form of malignant, lethal primary astrocytic, and a highly angiogenic brain tumor in humans’ central nervous system. It accounts for 12–15% of all intracranial tumors and 50–60% of all primary brain tumors [1,2]. The treatment of GBM is still unsatisfied due to its aggressiveness with poor prognosis, with a median survival of 14.6 months from diagnosis [3,4]. Treatments of GBM usually include radiotherapy alongside surgical resection and combined with chemotherapy such as temozolomide (TMZ), which is the landmark treatment protocol [5]. However, radiotherapy-induced alterations in the brain microenvironment may result in GBM recurrence and aggressiveness [6]. The survival rate for patients with glioblastoma remains dismal, and around >75% of GBM patients treated with TMZ succumb within 2 years due to relapse [7], and it also often triggers significant long-term debilitating side effects in survivors. Thus, finging a new compound for reducing side effects on GBM patients is urgent.

Many plant-based phytochemicals have been considered to be associated with human health, and they have even proved to reduce the risk of certain types of cancer [8,9,10,11,12]. Curcuminoids are natural and polyphenol coloring compounds derived from *Curcuma longa* Linn, which is also an edible plant. Curcuminoids contain three major bioactive ingredients: curcumin, demethoxycurcumin (DMC), and bisdemethoxycurcumin (BDMC), at a ratio of 77:17:3 [13]. The curcuminoids in the turmeric acetone extract are crucial for the gastroprotective effect against ethanol-induced damage [14]. BDMC prevented kidney fibrosis by activating fibroblast apoptosis [15] and promoted apoptosis in human platelets via activation of ERK signaling pathway [16]. It induced cell apoptosis in liver cancer Hep 3B cells [17] and induced caspase-dependent and -independent apoptosis via Smad or Akt signaling pathways in osteosarcoma HOS cells [18]. Furthermore, BDMC possessed favorable cardioprotection in an Nrf2/HO-1-dependent manner and activated the expression of Nrf2/HO-1 via PI3K/AKT signaling [19]. It also suppressed adipogenesis in 3T3-L1 adipocytes and prevented high-fat diet-induced obesity in mice [20]. Besides, BDMC attenuates cisplatin-induced renal injury through anti-apoptosis in renal tubular epithelial cells in vitro and protective effect on cisplatin-induced kidney injury in mice [21]. It was reported that oral treatment with micellar BDMC led to quantifiable concentrations of BDMC in glioblastoma patients that may alter intratumoral energy metabolism [22]. Recently, we combined all-trans retinoic acid (ATRA) with BDMC to treat human liver cancer Hep 3B cells, and the results led to increasing apoptotic cell death than that of ATRA or BDMC treatment only in vitro [17].

Although many studies have shown BDMC induced apoptotic cell death in many human cancer cells; however, there are no reports to show BDMC reduced brain tumor cells in the animal model in vivo. Therefore, in the present study, we used human glioblastoma GBM 8401 cell xenograft mice, and then the animals were treated with BDMC. We are the first to show BDMC suppressed tumor growth of GBM 8401 cell xenograft nude mice in vivo. These findings offer more information on BDMC, which may further consideration for future use in glioblastoma patients.

## 2. Results

### 2.1. BDMC Reduced Cell Viability, Induced Apoptosis, and Affected Apoptosis-Associated Proteins in GBM 8401/luc2 Cells

**Figure 1:**
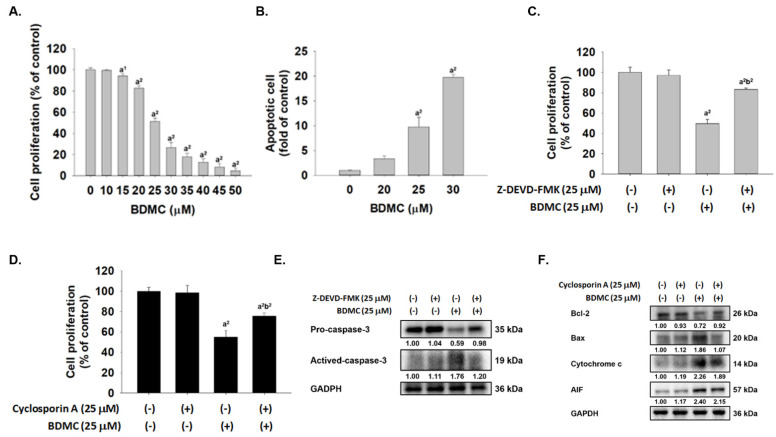
BDMC induced cytotoxic effect and cell apoptosis and affected apoptosis-associated protein expression in GBM 8401/*luc2* cells. Cells were placed in cell plates at the final density of 1 × 10^5^ cells/mL for 48 h, and cells were treated with 0, 10, 15, 20, 25, 30, 35, 40, 45, and 50 μM of BDMC for 48 h. After treatment, cells were harvested from each well for measuring cell proliferation (**A**) and apoptosis (**B**). Cells were pretreated with Z-DEVD-FMK (caspase-3 inhibitor) (**C**) and inhibitor of mitochondria membrane potential (cyclosporin A) (**D**) and were treated with 0 and 25 μM of BDMC for 48 h and cell proliferation were determined. Cells were harvested for examining apoptosis-associated protein expression (**E**,**F**) by western blotting as described in Materials and methods. a^1^ and a^2^ significantly different at *p* < 0.05 and *p* < 0.01 vs. the control group. b^2^ significantly different at *p* < 0.01 vs. the BDMC group.

MTT assay was used to measure cell cytotoxicity, and flow cytometer was used to assay cell apoptosis. After, GBM 8401/*luc2* cells were treated with various concentrations of BDMC for 48 h and cells assayed cell cytotoxicity and apoptosis, and results are shown in Figure 1A,B, which demonstrates that BDMC suppressed total viable cell number and the inhibitory rate was dose-dependently increased following BDMC treatment (*p* < 0.05). As presented in Figure 1A, after 48 h treatment, GBM 8401/*luc2* cell population was inhibited at 17.36–95.60% with 15–50 μM BDMC. Annexin V was used to quantify GBM 8401/*luc2* cell apoptosis after BDMC treatment, and results are shown in Figure 1B. After 48 h, BDMC treatment dose-dependently increased the cell apoptosis about 3–19 fold; thus, the cell apoptosis was significantly increased after treatment with BDMC compared with the control (*p* < 0.05). Cells were pretreated with or without Z-DEVD-FMK (caspase-3 inhibitor) or inhibitor of mitochondria membrane potential (cyclosporin A) and were treated with 0 and 25 μM of BDMC for 48 h. Cell proliferation was measured, and results were presented in Figure 1C,D. Figure 1C indicated that cells that were pretreated with Z-DEVD-FMK and then treated with BDMC experienced significantly increased cell proliferation up to 26% when compared to BDMC treatment alone. Results from Figure 1D also showed that cells were pretreated with cyclosporin A and then treated with BDMC significantly increased cell proliferation up to 14% when compared to BDMC treatment alone. Furthermore, BDMC decreased cell numbers and induced apoptosis were involved in apoptosis-associated protein, and cells were pretreated with or without Z-DEVD-FMK or cyclosporin A and then treated with 0 and 25 μM of BDMC for 48 h. Apoptosis-associated proteins were measured by western blotting, and results are presented in Figure 1E,F. Figure 1E indicates that the pretreatment of Z-DEVD-FMK and treated with BDMC led to an increase of pro-caspase-3 and decrease of active-caspase-3 when compared to BDMC treated only. Figure 1F indicates that the pretreatment of cyclosporine A and treated with BDMC led to an increase of Bcl-2 and decrease of Bax, cytochrome c and AIF when compared to BDMC treated only. Based on both results, BDMC induced cell apoptosis via affected apoptosis-associated proteins in GBM 8401/*luc2* cells

### 2.2. Flow Chart of In Vivo Experiments

**Figure 2:**
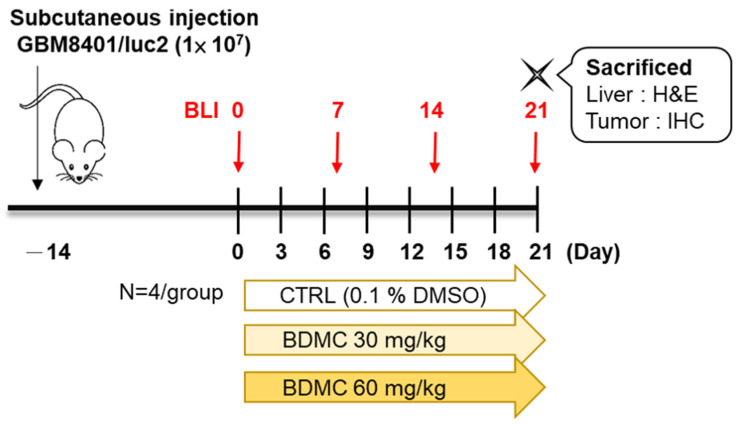
The flow chart for BDMC treatment in athymic BALB/c nu/nu nude mice after inoculation with GBM 8401/*luc2* cells. Twelve mice were inoculated subcutaneously with GBM 8401/*luc2* cells. Mice were randomly divided into three groups for treatment every day. Group I (*n* = 4) was treated 0.1% DMSO in 100 μL of *DDW*. Group II (*n* = 4) was treated with BDMC (30 mg/kg). Group III (*n* = 4) was treated with BDMC (60 mg/kg). Each mouse was treated by gavage for 21 days; the body weight and tumor size were recorded every 3 days, and bioluminescent imaging was performed every week, as described in the Materials and Methods.

The overall design of the experiments is presented in Figure 2, which shows the ingestion of GBM 8401/*luc2* cells and the treatment of BDMC and sacrificed animals.

### 2.3. BDMC Did Not Affect the Body Weight and Liver Pathology of Athymic BLAB/c nu/nu Nude Mice Bearing GBM 8401/luc2-Derived Tumors

**Figure 3:**
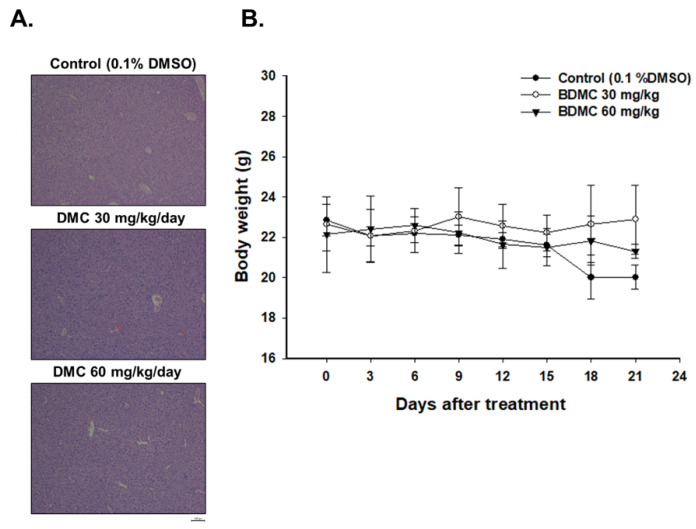
Liver H&E staining and body weights of athymic BALB/c nu/nu nude mice after inoculation with GBM 8401/*luc2* cells. (**A**) Bright view microscope image of liver H&E staining from each group (×100). (**B**) Mouse body weight was measured by digital balance every 3 days.

The results shown in Figure 3A indicate that treatment with BDMC at 30 and 60 mg/kg did not lead to liver pathology change as compared to the control group. Besides, in Figure 3B, no statistically significant differences in body weight of mice were found in the treated or control group. These results suggested that BDMC may not induce general toxicity of mice, including acute or delayed toxicity.

### 2.4. BDMC Inhibited GBM 8401/luc2 Cell Xenograft Tumor Growth in Nude Mice Bearing GBM 8401/luc2-Derived Tumors

**Figure 4:**
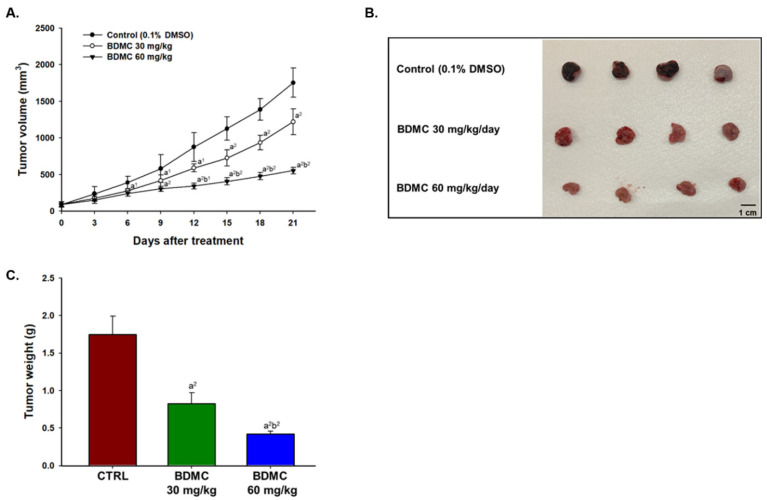
BDMC reduces GBM 8401/*luc2* cell xenograft tumor growth in athymic BALB/c nu/nu nude mice. After GBM 8401/*luc2* tumor-bearing mice were generated, mice were treated with DDW (0.1% DMSO) or BDMC (30 and 60 mg/kg) every three days. (**A**) The tumor volume of each mouse was measured every three days. (**B**) Images of representative mice tumors from each group. After mice were anesthetized with overdose isoflurane (>3%), they were sacrificed, and the final tumor weights were measured as described in the Materials and Methods. (**C**) Quantification results of mouse tumors weight from each group. a^1^ and a^2^ significantly differ at *p* < 0.05 and *p* < 0.01 vs. the control group. b^1^ and b^2^ significantly differ at *p* < 0.05 and *p* < 0.01 vs. the BDMC 30 mg/kg group.

Mice treated with 60 mg/kg BDMC exhibited significantly smaller tumor volume at day 6 (*p* < 0.05) and day 9–21 (*p* < 0.01) after the first treatment (Figure 4A). The representative tumor photographs with or without BDMC treatment were shown in Figure 4B, which indicated that BDMC treatment reduced the tumor size when compared to the control group. The higher dose of BDMC (60 mg/kg) has significantly reduced tumor weights than that of lower dose of BDMC (30 mg/kg) (*p* < 0.01) (Figure 4C). Taken together, BDMC showed the potential to inhibit tumor growth on glioblastoma bearing animal models.

### 2.5. BDMC Reduced Living Cell Signal of Nude Mice Bearing GBM 8401/luc2-Derived Tumors

**Figure 5:**
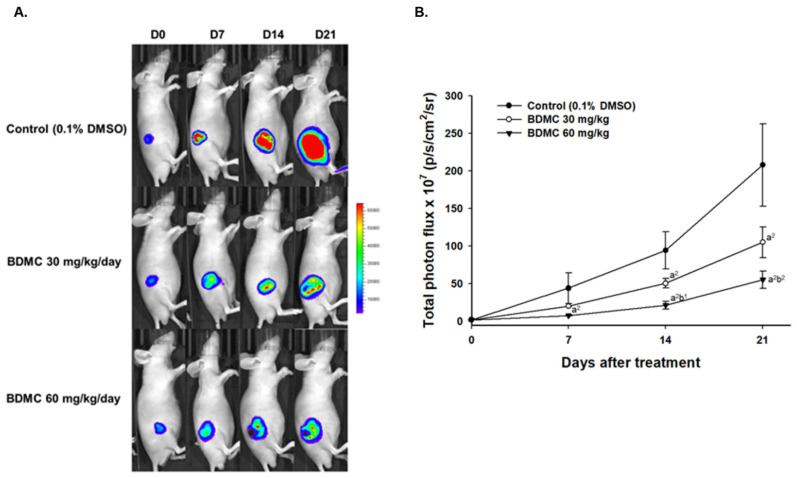
*Luc2* signals from living tumor cells were suppressed by BDMC treatment. (**A**) The representative BLI results from each group at different time points. (**B**) Quantification results of *luc2* signal intensity of tumors. (a^2^ *p* < 0.01 vs. control; b^1^ *p* < 0.05 and b^2^ *p* < 0.01 vs. BDMC 30 mg/kg).

To further investigate whether tumor growth inhibition was correlated to reduce the living cell population within the tumor, we performed the scanning of CMV-derived *luc2* reporter gene on GBM-8401/*luc2*-bearing tumor mice. As shown in BLI results from each group of mice, a relatively cold signal was found in the BDMC-treated groups on the color-coded map (Figure 5A). The *luc2* reporter gene intensity on the tumor was measured every week and quantified by Living Image software (v2.2). The total photon flux of the control group was two to four times more than that of BDMC-treated mice (Figure 5B). Significant living cell signal suppression was found in the higher dosage of the BDMC-treated group since day 7. In sum, BDMC treatment may reduce the living cell population within the tumor area.

### 2.6. BDMC Affects the Expression of Apoptosis-Related Protein Signal Pathway of Nude Mice Bearing GBM 8401/luc2-Derived Tumors

**Figure 6:**
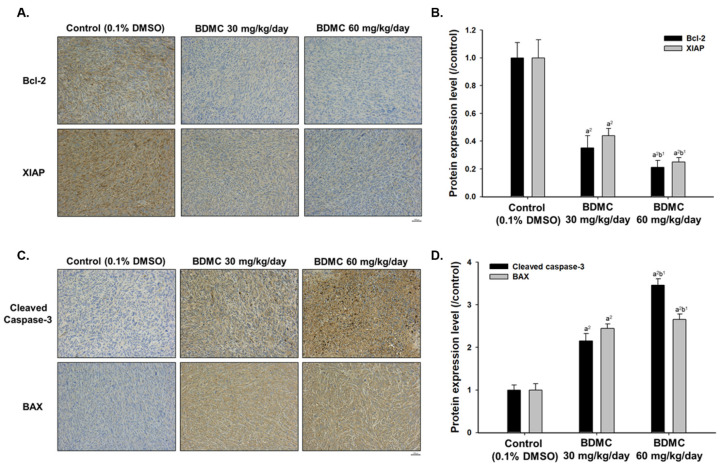
BDMC affects the expression of apoptosis-related proteins in GBM 8401/*luc2* cell xenografts. After treatment, tumors were isolated from GBM 8401/*luc2* xenografts, and IHC staining was performed as described in the Materials and Methods for Bcl-2 apoptosis regulator (Bcl-2) and X-linked inhibitor of apoptosis protein (XIAP) (**A**) and their levels of proteins (**B**); cleaved caspase-3 and Bcl-2-associated X (BAX) (**C**) and their levels of proteins (**D**). a^2^ significantly different at *p* < 0.01 vs. the control group. b^1^ significantly different at *p* < 0.05 vs. the BDMC 30 mg/kg group. Light brown color means the low expression of proteins and heavier brown colors means higher expression of proteins.

To fully evaluate the tumor inhibition mechanism of BDMC treatment on GBM 8401/*luc2* cell xenografts, tumors were collected from each animal to examine the expression of the apoptosis-associated protein by IHC staining. Results indicated that BDMC decreased the levels of Bcl-2 and XIAP (Figure 6A,B) and increased that of cleaved caspase-3 and BAX (Figure 6C,D). Overall, these data demonstrated that BDMC suppressed tumor properties in vivo via activation of apoptosis signaling.

### 2.7. BDMC Effectively Triggers the Apoptosis Mechanism and Suppressed Glioblastoma Tumor Growth

**Figure 7:**
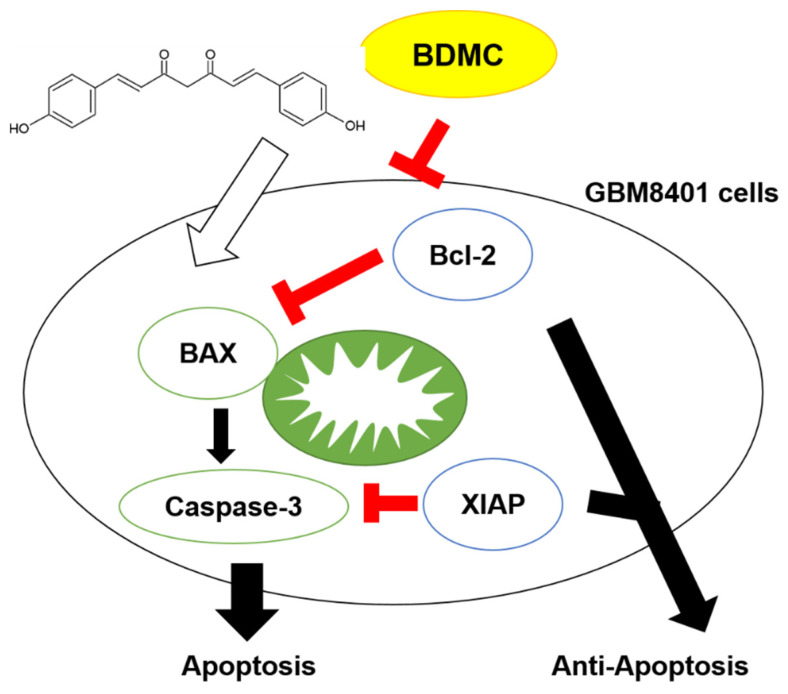
The mechanisms involved in inhibiting tumor growth upon BDMC treatment.

Both Bcl-2 apoptosis regulator (Bcl-2) and X-linked inhibitor of apoptosis protein (XIAP) were recognized as anti-apoptosis factors that contribute to tumor progression, while Bcl-2-associated X (BAX) and caspase-3 were known as apoptosis markers. In Figure 7, we demonstrated the potential mechanism for BDMC-induced inhibition of tumor progression is through blockage of Bcl-2 and XIAP and the promotion of BAX and caspase-3 cleavage in vivo.

## 3. Discussion

Currently, the standard therapy for high-grade glioblastomas (HGGs) (one of the most aggressive malignant primary brain tumors) such as GBM is radiotherapy [23], but the radio-resistance during the course of irradiation is one of the efficacy limitations [24,25]. The majority of HGGs eventually recur; thus, there are no effective treatments for this population. Therefore, intensive research and development of alternative effective anticancer drugs for patients with GBM is critical and needed [26].

Recently, studies have focused on pursuing a new therapeutic target in solid tumors, and this target is associated with poor survival among GBM patients [27,28,29]. Many plant-derived products (such as paclitaxel, vinblastine, and etoposide) and their derivatives have been recognized to be a standard repertoire of cancer chemotherapy [30]. Recently, it was reported that BDMC inhibited cell proliferation and induced cell apoptosis of GBM 8401 cells [31]; however, there are no reports to show BDMC how affected apoptosis-associated protein expression and affected the tumor growth of GBM 8401 cells in vivo. Therefore, we further investigated BDMC-induced cell apoptosis and affected apoptosis-associated protein expression, furthermore, to investigate the effects of BDMC on the tumor growth of GBM 8401 cells in subcutaneous nude mice in vivo.

In the present study, we investigated the cytotoxic effects of BDMC on GBM 8401/*luc2* cells in vitro, and results show that BDMC reduced total cell viability (Figure 1A) and induced cell apoptosis (Figure 1B) of GBM 8401/*luc2* cells, and that these effects were dose-dependent. These findings are in agreement with BDMC-induced cytotoxic effects and induced cell apoptosis in GBM 8401 cells [31]. However, apoptosis-associated protein expression, whether or not it was involved in BDMC-induced apoptosis in GBM 8401 cells are not available. Furthermore, it is well known that cell apoptosis may occur through caspase-3 or mitochondria dependently; thus, we also investigated BDMC regarding whether or not it affected caspase-3 or cytochrome c release in GBM 8401/*luc2* cells. Currently, results also showed that BDMC induced cell apoptosis via caspase-3 and mitochondria based on the co-treatment of Z-DEVD-FMK (caspase-3 inhibitor) or inhibitor of mitochondria membrane potential (cyclosporin A) and 0 and 25 μM of BDMC, leading to an increase of cell viability when compared to BDMC treated alone (Figure 1C,D). Moreover, the results (Figure 1E,F) from western blotting also indicated that BDMC decreased anti-apoptotic protein Bcl-2, increased pro-apoptotic protein BAX, and increased cytochrome c and AIF release in GBM 8401/*luc2* cells in vitro.

There are no reports to show BDMC inhibits tumor growth of GBM 8401/*luc2* cells in vivo; therefore, the primary purpose of this study is to evaluate the inhibition of tumor growth of BDMC in the animal model by different doses, times, and regimens. We used human glioblastoma GBM 8401/*luc2* cell-generated xenograft mice model for in vivo experiments, and our results indicated that BDMC at both doses (30 and 60 mg/kg) treatment significantly suppressed the tumor volume and weight of GBM 8401/*luc2*-cell-generated xenograft mice in vivo (Figure 3). Furthermore, BDMC elevated the active form of caspase-3 and pro-apoptotic protein BAX and decreased the anti-apoptotic protein Bcl-2 and XIAP (Figure 6).

The preclinical model, which is based on patient-derived tumor xenografts, has new insight into many clinical fields; thus, many authors have recognized immunodeficient animals such as athymic rats and mice to be used to prevent tissue loss caused by acute rejection to establish patient-derived tumor xenografts models. It is well documented that athymic nude mice were suitable for examining natural compounds or anticancer drugs that inhibit cancer cell-generated xenograft mice model in vivo [32]. Recently, in our earlier studies, we established GBM 8401/*luc2* cell xenograft tumor growth in athymic BALB/c nu/nu nude mice for evaluating whether or not compounds such as BITC and PEITC reduced tumor in vivo [33,34]. A similar animal model (GBM 8401/*luc2* cell xenograft mice) was also used in this study. The body weights of tumor-bearing mice in all groups were simultaneously recorded. The BDMC-treated and control group did not show differences in the body weight during treatment period, indicating no systemic toxicity of BDMC (Figure 3).

Results indicated that significant difference of tumor size was observed between BDMC-treated and control groups. The control group showed a progressive increase in tumor volume, while tumors in both BDMC-treated groups displayed growth retardation. BDMC decreased tumor volume and weight in GBM 8401/*luc2* cell xenograft mice, and a higher dose (60 mg/kg) of BDMC treatment has a higher inhibition than that of a lower dose (30 mg/kg) (Figure 4). Furthermore, we also used this CMV-derived *luc2* reporter gene to monitor the tumor growth of GBM 8401/*luc2*-bearing tumor mice. As shown in BLI results from each group of mice, a relatively cold signal was found in the BDMC-treated groups on the color-coded map (Figure 5A). The total photon flux of the control group was two to four times more than BDMC-treated mice (Figure 5B). Significant living cell signal suppression was found in higher dosage (60 mg/kg) of BDMC-treated mice since day 7. BDMC treatment may reduce the living cell number within the tumor area. This finding is consistent with the results of tumor size and weights (Figure 4).

It is recognized that the compounds could be suitable as tumor-inhibiting agents with the ability to simultaneously block multiple hallmarks of tumor capabilities [35], specifically in inducing cancer cell apoptosis without causing cellular toxicity. In this study, we isolated the liver from each mouse of each group; livers were performed with H&E staining, and results show that BDMC did not induce toxicity in animals (Figure 3A). If an agent can alter protein expression or simultaneously block multiple cancer-associated pathways, it will be developed for a viable approach to inhibit tumor growth and progression. Therefore, to further confirm BDMC displayed in tumor growth retardation compared to control groups, herein, we also took tumor samples from each mouse of each group for examining apoptosis-associated protein expression by BDMC treatment. Our results indicated that tumor tissues of mice exposed to BDMC showed relatively lower levels of anti-apoptotic proteins (Bcl-2) and XIAP when compared to the control group. However, tumor tissues of mice exposed to BDMC showed higher levels of cleaved caspase-3 and BAX (pro-apoptotic protein) (Figure 6). Significant differences were observed between BDMC-treated and control groups (*p* < 0.01), clearly indicating that BDMC is considerably effective in inducing apoptosis and inhibiting tumor growth (Figure 6).

Bcl-2 family proteins, which are divided into anti-apoptotic proteins, such as Bcl-2, and pro-apoptotic proteins such as BAX, are involved in cell apoptosis. The decreases of the Bcl-2/BAX ratio lead to the release of cytochrome C from the mitochondria for activating the mitochondrial-dependent caspase cascade and then inducing apoptotic cell death [36,37]. In the mitochondrial membrane, BAX is a constituent of the ion channel involved in the loss of Δψm. Bcl-2 is an oncogene with multiple anti-apoptotic functions, and it combines with BAX to prevent the formation of the ion channel [38]. Therefore, it is well documented that the Bcl-2/BAX ratio acts as a critical role in the induction of apoptosis. In the present study, BDMC significantly inhibited the tumor growth of GBM 8401 xenograft mice in vivo via upregulating the expression of BAX (pro-apoptotic factor) and caspase-3 and downregulating the expression of Bcl-2 (anti-apoptotic factor) and XIAP in tumor tissues. It is well known that increasing cell apoptosis of the tumor in the therapeutic process may result in a reduction of tumor size [39].

The apoptosis caspases (a cysteine protease family) and the apoptosis suppressor gene Bcl-2 (Bcl-2 family) both play important roles in the apoptosis process. Caspase can activate the whole protease family after itself activation [40,41]. The caspases-3 is one of the caspase family proteins and exerts a vital apoptosis effector, which can be activated by the upstream initiating subsystem, or via caspase-9 to make the cell biochemical changes and morphological changes, and then finally lead to cell apoptosis [42,43]. The X-linked inhibitor of apoptosis (XIAP), a potent inhibitor of cell death, exerts the ability to directly suppress the caspases [44] when the interaction of caspase 9 and 3 allows the XIAP to block the caspases activities efficiently, preventing a proteolytic cascade. Recently, literature has reported higher expressions of XIAP and Bcl-2 in the GBM samples than that of normal brain samples and XIAP involved in apoptosis resistance [45]; thus, XIAP has been recognized to be the best way to develop new target therapy strategies among GBM patients.

In conclusion, overall, in the present study, in vivo experiments using a GBM 8401/*luc2* cell xenograft tumor growth in athymic BALB/c nu/nu nude mice model showed that BDMC treatment significantly decreased the tumor volume and weight compared to control groups. Moreover, the body weight and liver tissues in H&E staining from each mouse of each group showed that the BDMC treatment did not cause significant systemic toxicity to the mice. Tumor tissues were stained with apoptosis-associated antibody, which indicated that BDMC-induced cell apoptosis. Thus, we suggested that BDMC may act as an effective chemosensitizer against GBM patients in the future.

## 4. Materials and Methods

### 4.1. Test Compound, Reagents, Antibodies, and Culture Medium

Bisdemethoxycurcumin (BDMC) was purchased from ChemFaces (Wuhan, Hubei, China) and dissolved in dimethyl sulfoxide (DMSO) as 150 mg/mL stock (Sigma Chemical Co., St. Louis, MI, USA). Roswell Park Memorial Institute (RPMI) 1640 Medium, fetal bovine serum (FBS), and penicillin-streptomycin were purchased from Life Technologies (Carlsbad, CA, USA). JetPEI™ transfection reagent was obtained from Polyplus Transfection (Illkirch, Bas-Rhin, France). D-luciferin and pGL4.50 luciferase reporter (pGL4.50[*luc2*/CMV]) vector were obtained from Promega (Madison, WI, USA). Hygromycin B was obtained from Santa Cruz Biotechnology (Santa Cruz, CA, USA). Primary monoclonal antibody for immunohistochemistry staining was listed as follows: anti-Bcl-2 (1:300 dilution; Cell signaling, Danvers, MA, USA), anti-XIAP (1:300 dilution; Elabscience Biotechnology Inc., Houston, TX, USA), anti-cleaved caspase-3 (1:300 dilution; Cell signaling), and anti-BAX antibodies (1:300 dilution; Rosemont, IL, USA).

### 4.2. Cell Culture of GBM 8401 Cells

Human brain glioblastoma cell line (GBM 8401) was obtained from The Food Industry Research and Development Institute (Hsinchu, Taiwan) and was cultured in RPMI-1640 supplemented with 10% FBS, 100 U/mL penicillin, and 100 µg/mL streptomycin in 5% CO_2_ humidified incubators at 37 °C [34].

### 4.3. Cell Culture, Transfection, and Stable Clone Selection

Human glioblastoma GBM 8401 cells were cultured in the 10-cm dish with RPMI-1640 supplemented with 10% FBS, 100 U/mL penicillin, and 100 µg/mL streptomycin, in 5% CO_2_ humidified incubators at 37 °C [34]. Plasmid transfection and stable clone selection protocol were described in our previous studies [46,47]. In brief, pGL4.50 luciferase reporter (pGL4.50[*luc2*/CMV]) plasmid and JetPEI™ transfection reagents mixture was added to GBM 8401 cells overnight. Then, 200 μg/mL hygromycin B was used to select and maintain the stability of *luc2* expression in GBM 8401 cells. *Luc2* signaling was acquired by IVIS 200 Imaging System (Xenogen, Alameda, CA, USA). This stable *luc2* reporter gene cell was defined as GBM 8401/*luc2*.

### 4.4. Measurements of Cytotoxicity

Cell cytotoxicity (cell viability) was determined by MTT assay. GBM 8401/*luc2* cells (1 × 10^4^ cells/well) were placed in 96-well plates for 24 h and then were treated with 0, 10, 15, 20, 25, 30, 35, 40, 45, and 50 μM of BDMC for 48 h. In brief, a 10 μL solution (5 mg/mL MTT) was added to each well for 4 h at 37 °C; discarded supernatant, followed a 100 μL DMSO, was added to well for dissolving the purple formazan crystalas described previously [48]. All treatment concentrations were performed in three independent tests.

### 4.5. Annexin V/PI Staining for Cell Apoptosis Assay

The percentage of cell apoptosis was evaluated by using the Annexin V-FITC/PI Apoptosis Detection kit followed the guideline from the manufacturer. GBM 8401/*luc2* cells were seeded into 24-well tissue culture plates (1 × 10^5^ cells/well) for 24 h and were treated with 0, 20, 25, and 30 μM of BDMC for 48 h. Following treatment, the cells were collected, washed with PBS, and resuspended in 200 μL Annexin V binding buffer and were incubated with Annexin V-FITC/PI in the dark for 15 min and samples were analyzed of cell apoptosis by flow cytometry (BD FACSCanto, Franklin Lakes, NJ, USA) as described previously [49].

### 4.6. Cytoxicity of Cotreatment BDMC with Caspase-3 or MMP Inhibitor

GBM 8401/*luc2* cells (1 × 10^4^ cells/well) were placed in 96-well plates for 24 h and then were pretreated with Z-DEVD-FMK (caspase-3 inhibitor) (25 μM) or inhibitor of mitochondria membrane potential (cyclosporin A) (1 μM) and were treated with 0 and 25 μM of BDMC for 48 h. Cells proliferation (viability) was measured by MTT assay as described previously [48]. All experiments were done in triplicate.

### 4.7. Western Blotting

GBM 8401/*luc2* cells (1.5 × 10^6^ cells/dish) were placed in 10-cm dish and pretreated with Z-DEVD-FMK (caspase-3 inhibitor) (25 μM) or inhibitor of mitochondria membrane potential (cyclosporin A) (1 μM) and were treated with 0 and 25 μM of BDMC for 48 h. All cells from each treatment were collected, extracted, and quantitated total proteins using the Bio-Rad Protein Assay kit (Bio-Rad, Hercules, CA, USA). A 30 μg of sample was separated by 10% sodium dodecyl sulfate-polyacrylamide gel electrophoresis and was transferred onto polyvinylidene difluoride membranes (Millipore, Billerica, MA, USA). All membranes were incubated with primary antibodies (ant-pro-caspase-3, -active-caspase-3, -Bcl-2, -Bax, -cytochrome c, and -AIF) overnight, washed, and then incubated with peroxidase-labelled secondary antibody. The membranes were washed and incubated in ECL western blot analysis substrate (Bio-Rad, Hercules, CA, USA), and the protein bands were visualized and photographed as described previously [48]. All results were obtained from three independent experiments.

### 4.8. Establishment of Glioblastoma Xenograft Bearing Mice and Treatments

Athymic BALB/c nu/nu (NUDE mice), ~22–28 g in weight and 6–8 weeks of age, were obtained from the National Laboratory Animal Center (Taipei, Taiwan). Mice were maintained at a constant temperature of 25 ± 1 °C in 55% humidity with a 12 h light/dark cycle and were regularly provided with food and water. All animal experiment procedures had been approved by the institutional animal care and use committee (IACUC) in China Medical University, Taichung, Taiwan (ID: CMU2019–204). GBM 8401/*luc2* cells (1 × 10^7^ cells in 150 μL serum-free RPMI-1640 and Matrigel (2:1) mixture) were subcutaneously injected into the right flanks of the mice, and two weeks later, the mice were successfully induced as a model of glioblastoma [33,50]. A total of 12 mice were randomly divided into three groups (*n* = 4 each) when the tumor reached 100 mm^3^ and subsequently received different treatments as described below. Group I mice served as control and were treated with 0.1% DMSO, which dissolved in 100 μL double-distilled water (DDW) for 21 days. Group II and III were treated with 30 mg/kg and 60 mg/kg BDMC for 21 days, respectively (both dissolved in 100 μL DDW) (Figure 2).

### 4.9. Treatment Efficacy Evaluation and Animal Bioluminescent Imaging

Mice tumor size and body weight were evaluated by caliper (Mitutoyo America, Aurora, IL, USA) every three days. Tumor volumes were measured by the formula: volume (mm^3^) = (0.523) × length (mm) × width^2^ (mm) [33]. In vivo bioluminescent imaging (BLI) was performed on days 0, 7, 14, and 21 after BDMC treatment. Mouse tumor from each group was isolated and weighed on day 21 and then fixed for further H&E and IHC staining (Figure 2). 100 μL D-luciferin (150 mg/kg dissolved in PBS) was injected into the peritoneal by a 30-gauge needle 15 min before image acquisition. After 15 min, mice were anesthetized with 1–3% isoflurane and scanned by IVIS^®^ Lumina LT Series III (Bruker, Billerica, MA, USA) for 3 min. The signal intensities of BLI were quantified by using Living Image software (Version 2.20, Xenogen, Alameda, CA, USA).

### 4.10. Hematoxylin and Eosin (H&E) and Immunohistochemistry (IHC)

At the end of treatment, liver and tumor tissues were isolated and fixed in 10% neutral buffered formalin for 24 h and embedded in paraffin. Paraffin-embedded liver tissues were sliced at 5 μm, deparaffinized, rehydrated, and stained with hematoxylin and eosin (H&E) [51]. Paraffin-embedded tumor tissues were also sliced at 5 μm; deparaffinized; subjected to antigen retrieval; and stained with anti-Bcl-2, anti-XIAP, anti-cleaved caspase-3, and anti-BAX primary antibodies at 1:300 concentrations. After 24 h antibodies incubation at 4 °C, secondary antibodies were incubated for another 1 h then washed and incubated with Horseradish Peroxidase Streptavidin (HRP Streptavidin) as protocol described in EMD Millipore’s IHC Select^®^ kit (EMD Millipore, Billerica, MA, USA) [52]. Sections were finally rinsed by distilled water, dehydrated, and mounted with mounting medium for Nikon ECLIPSE Ti-U light microscope evaluation (Nikon Instruments Inc., Melville, NY, USA). Image of IHC was quantified by ImageJ software version 1.50 (National Institutes of Health, Bethesda, MD, USA).

### 4.11. Statistical Analysis

All data represent as mean ± S.D. In vitro study, a significant difference was compared by one-way ANOVA. In vivo study, a significant difference between the BDMC 60 mg/kg-treated, BDMC 30 mg/kg-treated and control groups were compared by Student’s *t*-test. In all figures, significant difference was presented as a^1^ *p* < 0.05 or a^2^ *p* < 0.01 vs. control; and b^1^ *p* < 0.05 or b^2^ *p* < 0.01 vs. BDMC-treated.

## Data Availability

Data available on request from corresponding author.
